# Expectations of Health Researchers From Academic Social Network Sites: Qualitative Study

**DOI:** 10.2196/24643

**Published:** 2021-12-07

**Authors:** Mohammad Dehghani, Mehdi Kahouei, Shahin Akhondzadeh, Bita Mesgarpour, Reza Ferdousi

**Affiliations:** 1 Department of Health Information Technology Khomein University of Medical Sciences Khomein Iran; 2 Social Determinants of Health Research Center School of Allied Medical Sciences Semnan University of Medical Sciences Semnan Iran; 3 Psychiatric Research Center Roozbeh Hospital Tehran University of Medical Sciences Tehran Iran; 4 National Institute for Medical Research Development (NIMAD) Tehran Iran; 5 Department of Health Information Management School of Management and Medical Informatics Tabriz University of Medical Sciences Tabriz Iran

**Keywords:** research, social network, academic social network, research network, academic, researcher, literature, qualitative, content analysis

## Abstract

**Background:**

Today, academic social network sites' role in improving the quality of education and how investigators conduct their research has become more critical.

**Objective:**

This study aimed to investigate Iranian health researchers' requirements for academic social network sites from a low-income country perspective.

**Methods:**

This qualitative study with a phenomenological approach was done in 2020. In this study, 23 researchers in the health system were selected by purposive sampling. Semistructured interviews were used to collect data. Data were analyzed by MaxQDA-10 software and the content analysis method.

**Results:**

We identified 2 categories of functional and technical characteristics in the study participants' expectations. Functional characteristics included facilitating communication and team activities, managing scientific publications, enhancing the process of conducting research, being informative, and sharing and trading laboratory materials and equipment. Technical characteristics of an academic social network include user management capabilities, high security and privacy, being user-friendly, and other technical features.

**Conclusions:**

Health researchers emphasized 2 functional and technical characteristics required to meet academic social network sites' expectations.

## Introduction

Nonacademic social networking sites such as Facebook are prevalent, and researchers can use them. However, studies show challenges and restrictions for academic users on these sites [1-4]. Today, academic social network sites (ASNSs) have become an integral part of researcher work [5,6]. An ASNS is a type of internet service that facilitates communication between researchers [7], shares scientific resources (news, reports, articles, and data sets), exchanges research opinions, and informs about the current research trend [8]. In addition to publishing researchers' work and facilitating personal exchanges, ASNSs are tools for describing organizational information and researcher interests [9].

Ijad Madisch, one of the creators of Research Gate, acknowledges that ASNS promotes transparency in the research process and ultimately leads to the strengthening of scientific research [10]. In May 2017, Alexa.com ranked globally Research Gate and Academia.edu 321st and 577th, respectively, indicating increased use of ASNSs [11]. Based on Dong [12], ASNSs have a positive impact on the performance of academics. However, Salvation's study [13] in Malaysia found hidden weaknesses of ASNS.

Every ASNSs is customized for one or more specific purposes; for example, Research Gate is primarily for contacting colleagues and counseling. Mendeley offers the opportunity to receive new articles [14].

Along with researchers from other countries, Iranian health researchers use different ASNSs to conduct their research activities [15]. However, 61% of Iranian researchers do not trust this social network [16]. Ghazimirsaeed [17] examined the use of the academic social networks in Iran and showed 83% (44/53) of Iranian medical science universities were present in the ASNS in 2017. On average, 180 researchers from each university and 1161 departments of the medical universities were members of these ASNSs [17].

ASNSs are created professionally and with a specific purpose, and each of them has its particular users [18]. Investigating the needs and expects of researchers from ASNSs can increase the use of these social networks and make them successful. This study aimed to investigate health researchers' requirements from ASNS in Iran, as a low-income country. In addition to being used in the design of ASNS, this study's results can strengthen them.

## Methods

### Study Design

This qualitative study to identify Iranian researchers' expectations from ASNS was done in 2020. This article is excerpted from a doctoral dissertation entitled “Designing and Implementing a Social Network for Laboratory Researchers in Health” [19]. We chose the qualitative method to highlight participants' experiences, knowledge, and silent information [20,21]. We selected the phenomenological approach due to the lack of a complete theory of the expectations of Iranian researchers from ASNS [22].

### Participants and Setting

The study environment was a research center affiliated with medical colleges. Research managers, faculty members, postdoctoral researchers, and PhD students participated in this study. The inclusion criteria were the membership in 2 or more academic social networks and updating their user profile on academic social networks at least once a month. Participants were selected by the purposive sampling method. Individuals with good information and who provided their information appropriately were chosen as participants in this method [23-25]. The interviews with participants continued until information saturation was achieved, and researchers felt that new information on new participants was not available; this step was achieved with 23 participants. After the study's initiation and interviews, we used theoretical sampling to identify people who could provide rich and beneficial information for researchers.

We attempted to have a diversity of age, employment status, work experience, degree, and job position in our study sample.

### Data Collection

Semistructured interviews in the Persian language have been done conveniently for participants. The interview questions were developed by using literature reviews and expert opinions and comprised of five questions. The interviewee was initially asked to introduce themselves and explain their recent research activities. In the second question, the researcher was asked which academic social networks they use and why; and which features are interesting to them.

The next question was about the advantages and disadvantages of these social networks. In that question, participants were asked to compare 2 or more academic social networks they have used. The fourth question asked how academic social networks could accelerate the research process and improve their quality, and the final question queried which features would be considered if the researcher were to design an ASNS.

We used S-recorder software (version 20.1.186.12; Samsung) to record the interviews alongside note-taking. Each interview lasted between 30 and 42 minutes.

### Qualitative Analysis

The respondents' answers were immediately typed, summarized, and reviewed several times by listening to and reading the primary information.

Conventional content-method and MaxQDA-10 software (version 10; VERBI) were used for data analysis. The conventional content method is very useful for identifying, analyzing, and reporting the patterns (themes) in qualitative studies [26-28]. Respondent validity and immersed expert and peer checks were used for data portability, rigor, and reliability.

Following informed consent procedures, the research participants are provided with a brief verbal explanation of the study and told that they could leave the study at any time. The participant code and their job were used to report their statements to keep the information confidential. The ethics committee at Tabriz University of Medical Sciences approved this study (IR.TBZEDMED.REC.1398.184).

## Results

### Overview

**Figure 1:**
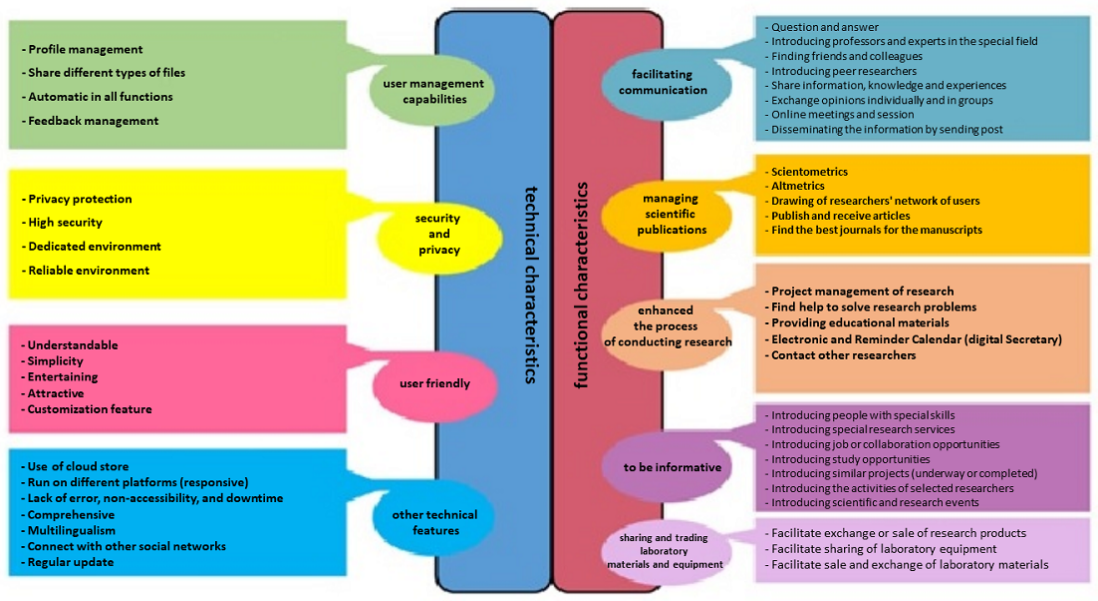
Health researchers' expectations for academic social network sites.

Study participants included 7 research managers, 6 faculty members, 4 postdoctoral researchers, and 6 PhD students. Based on the interviews, the researchers' expectations from the ASNSs were divided into 2 general categories: the system's functional and technical characteristics (Figure 1).

### Functional Characteristics

Functional characteristics included facilitating communication and team activities, managing scientific publications, enhancing the process of conducting research, being informative, and sharing and trading laboratory materials and equipment. Functional characteristics have 5 main themes and 29 subthemes. Based on the number of repetitions of the subtheme in the interviews, health researchers' most important expectation from ASNSs was to contact other researchers. Finding help to solve research problems, contacting other researchers, and introducing the activities of selected researchers are essential ASNS expectations for PhD students (Table 1).

According to the participants' scientific rank, the four columns of Tables 1 and 2 specify how many people have mentioned the relevant theme in their speeches. Moreover, the total number of people who have mentioned a particular theme is specified in the last column of the tables.

**Table 1:** Functional expectations of health researchers from academic social network sites.

Theme and subthemes		Description	PhD student (n=6)	Postdoctoral researcher (n=4)	Faculty member (n=6)	Research manager (n=7)	All (n=23)
**Facilitating** **communication and team activities**							
	Question and answer	Health researchers need to receive advice from experts through ASNS to solve their problems.	3	2	3	1	9
	Introducing professors and experts in the special field	Health researchers would like to get acquainted with the best professors and experts in their research field using ASNS.	2	1	4	0	8
	Finding friends and colleagues	ASNS must allow its users to be notified of their colleagues' membership in the social network based on their email or phone contact list.	4	1	4	2	11
	Introducing peer researchers with the same background	ASNS should introduce researchers with similar research backgrounds to each other based on information entered by users.	1	3	4	2	9
	Share information, knowledge, and experiences	The sharing of knowledge and experiences of researchers by the ASNS is critical and considered a requirement for the ASNS’s success.	0	2	5	3	10
	Exchange opinions individually and in groups	Dissemination of health researchers' in-network opinions and posts to individuals and groups will improve the quality of research.	0	0	5	3	8
	Online meetings and session	Although there are several specialized software for online meetings, it seems that the integration and access to more features, such as online meetings, can increase researchers' desire to use ASNSs.	3	1	3	4	11
	Disseminating the information by sending posts	Health researchers tended to publish their advertisements, comments, requests, and requirements by sending a post.	3	2	3	4	12
**Managing scientific publications**							
	Scientometrics	Researchers like to use this ASNS to monitor their citations.	2	3	5	4	14
	Altmetrics	This network feature shows how many times a document has been downloaded or read, And this can show how important and practical it is.	2	3	5	4	14
	Drawing of researchers' network	Showing researcher followers and followers has many advantages for other users. This allows them to get to know another person working in a common field.	2	1	2	1	6
	Publish and receive articles	The possibility of publishing and receiving articles is one of the essential parts of ASNS based on health researchers' views.	3	4	5	3	15
	Find the best journals for the manuscripts	The ASNS can introduce appropriate journals to users based on user profile data.	3	0	0	1	4
**Enhanced the process of conducting** **research**							
	Projects management of research	In some cases, health researchers have several responsibilities other than research, including education, patient care, and executive activities. Therefore, providing services via ASNS to manage research projects is very helpful.	0	1	2	3	6
	Find help to solve research problems	Various challenges, such as financial, administrative, property rights, laws, and access to protocols, are treated by health researchers during the research stage. As a facilitator, ASNS can play an essential role in solving these problems.	5	2	4	4	15
	Providing educational materials	Given that the research is based on innovations and problem solving, new research techniques and methods can be made available to researchers through the ASNS.	3	2	1	1	7
	Electronic and reminder calendar (digital Secretary)	Time management is one of the basic principles of research success; ASNS can play an essential role in managing researchers' time by providing tools such as electronic calendars and reminders.	0	1	1	0	2
	Contact other researchers	Creating different communication platforms by the network and facilitating communication between researchers promotes cooperation.	5	3	5	4	17
**To be** **informative**							
	Introducing people with special skills	When a researcher needs an expert with unique skills, the ASNS should support them with search tools.	4	2	3	1	10
	Introducing special research services	Researchers have different knowledge and skills. Sometimes they need laboratory services that are not available in their work environment. The research manager can inform you about the services available in a laboratory and share them with other researchers using an ASNS.	1	0	0	4	5
	Introducing job or collaboration opportunities	Health researchers can use ASNS to find jobs or collaboration opportunities in research projects.	1	3	4	0	8
	Introducing study opportunities	Providing study opportunities on ASNS and creating transparency can lead to a better selection of candidates.	0	2	4	3	9
	Introducing ongoing or completed similar projects	Searching for similar research projects and preventing duplicate works can provide good opportunities for collaboration between researchers.	0	3	5	1	9
	Introducing the activities of selected researchers	The following possibility of the researcher's activities allows users to obtain information about the latest scientific achievements.	5	2	4	3	14
	Introducing scientific and research events	Health researchers can use ASNSs to find out the news related to scientific and research events.	1	3	5	4	13
**Sharing and trading laboratory materials and equipment**							
	Facilitate to exchange or sale of research products	Researchers can share, exchange, or sell their research products on ASNSs.	1	2	2	5	10
	Facilitate to sharing of laboratory equipment	By creating the necessary content for sharing laboratory equipment, the ASNS can increase this laboratory equipment's efficiency and provide the opportunity for cooperation between research centers.	4	3	4	5	16
	Facilitate to sale and exchange of laboratory materials	In addition to reducing research costs, the exchange of laboratory materials among researchers accelerates research processes.	4	3	3	4	15

**Table 2:** Technical expectations of health researchers from academic social network sites.

Theme and subthemes		Description	PhD student (n=6)	Postdoctoral researcher (n=4)	Faculty member (n=6)	Research manager (n=7)	All (n=23)
**User management capabilities**							
	Profile management	Creating a user profile is the main feature of academic social networks.	5	3	5	6	19
	Share different types of files	Due to the production of knowledge in various formats by health researchers and the communication between them, sharing files in different formats is one of the essential features of the ASNS.	4	4	5	3	16
	Automatic in all functions	The automation of ASNS in different functions, such as informing and introducing colleagues, is one of these networks' main strategies to attract researchers.	2	1	1	3	7
	Feedbacks management	Posting comments on the ASNS about the researcher's activities in various forms such as text, confirm, and like can improve the researcher's activities. However, the user must manage the type and content of this feedback.	1	0	0	2	3
**High security and privacy**							
	Privacy protection	The most critical concern for health researchers in using ASNS is to protect their privacy.	2	4	5	5	16
	High security	Health researchers have become more sensitive to academic and social networks' security.	2	4	4	6	16
	Dedicated environment	ASNS' environment should be dedicated to researchers.	0	1	0	3	4
	Reliable environment	Researchers need a reliable social media environment to share scientific discussions and share their social media views.	0	2	2	3	7
**User friendly**							
	Understandable	The use of abstract and incomprehensible words in ASNS makes the social network unacceptable for researchers.	2	1	5	3	11
	Simplicity	Researchers should simply be able to use ASNS.	2	2	3	1	7
	Entertaining	The art of embedding gamification in the academic social network makes researchers more inclined to use this in their daily activities.	3	1	1	1	6
	Attractive	Observance of clarity and transparency, visual appeal, color selection, and visuals are criteria for making the ASNS more attractive.	3	2	2	4	11
	Customization feature	ASNS does not have wholly fixed characteristics, but a number of its features should be changeable based on the choice and needs of the researcher.	1	2	5	3	11
**Other technical features**							
	Use of cloud store	Today, cloud storage has become an essential requirement for researchers.	2	1	1	1	5
	Run on different platforms (responsive)	In the design process of a social network, all pages must be visible on all devices with similar content, design, and performance.	4	3	4	5	16
	Lack of error, non-accessibility, and downtime	The high rate of unavailability of the ASNS can reduce its users.	0	1	1	2	4
	Comprehensive	The comprehensiveness and non-allocation of the site to a group of researchers lead to researchers' rapid growth.	1	1	0	0	2
	Multilingualism	The multilingual aspect of ASNSs, in addition to understanding, can make a social network more trustworthy and more inclusive.	4	1	1	2	8
	Connect with other social networks	Communicating and retrieving information from other academic social networks, in addition to saving time, can make social networks more attractive.	1	2	5	5	13
	Regular update	In addition to updating security and information technology, ASNS should create new academic social network functions.	3	1	2	1	7

### Technical Features

The technical features are related to the social network's design, language, and the databases and infrastructure used to implement the social network. Based on the views of the study, participants' technical features consist of 4 main themes and 20 subthemes. However, the most important subtheme based on participant study views was profile management, but their concerns about security and privacy were considerable (Table 2).

## Discussion

### Principal Findings

This study aimed to identify Iranian health researchers' expectations for ASNS from the perspective of a low-income country.

One of the most important expectations of ASNSs was to create a platform for communication and to strengthen researchers' team activities. In line with this study's results, Salahshour [29] showed that 54% of researchers use ASNSs to find colleagues, and 75% of them use ASNS to communicate.  Krause [7] argued that in addition to creating intraorganizational communication, ASNSs should facilitate communication and the sharing of resources between scientists. Manca [30] also considers the most important task of ASNSs as establishing a relationship between researchers in the same field. Given that the study participants were researchers from a low-income country, they may have felt a greater need to connect and collaborate with other researchers in high-income countries.

Another functional expectation from ASNSs was the management of publication researchers' work. In line with the results of this study, several studies confirm that publishing management is an essential duty of ASNSs [31-33]. Salahshour [29] also found that 67 percent of users use ASNSs to improve citations and scientific advances. Weber [34] attributes researchers uploading the research results to ASNSs due to increased citations and establishing cooperation and communication between researchers [34]. However, Bonaiuti [9] attributes this behavior to the possibility of receiving feedback and the ease of loading articles in the ASNSs [9]. Because scientometric and altimetric indicators are among the main criteria for evaluating and ranking researchers, health researchers use ASNSs as a tool to display their articles. They try to improve the desired indicators by making their works available. Completing the profile correctly and updating the uploaded items plays a vital role in the researcher being seen by other colleagues. In addition to upgrading the altimetric rankings, ASNSs can improve the number of citations by creating communication capacities and collaborating with traditional metrics.

The researchers' third functional feature involved in the study was to help facilitate research and solve research projects by ASNSs. In a prior study, 56 percent of researchers said their goal for being a member of an ASNS was to improve research quality and learning [29]. In this regard, Espinoza [35] acknowledges that by creating communication, collaboration, and networking platforms, ASNSs support researchers and academics. Various studies have shown that ASNSs, in addition to their tools for communication, collaboration, question and answer, specialized discussion groups, and ability to introduce researchers with the same background, can support researchers and improve research quality [9,34,36]. The advantages of cooperation between researchers include reducing researchers' workload, regulating the activities of researchers based on expertise and skills, increasing the credibility and quality of research, increasing the number of studies, and increasing the productivity and efficiency of researchers. By creating a platform for communication and cooperation on the one hand and maintaining individuals' privacy, on the other hand, ASNSs provide the foundation for the cooperation and facilitation of research. One of the future challenges of ASNSs seems to be managing collaborations between researchers, managing collaboration requests, and protecting researchers' privacy.

Being informative is an essential expectation for ASNSs. Researchers believe, given their interest, an ASNS should automatically inform them of study opportunities, suitable jobs, and cooperation suggestions.  Findings from Dermentzi [37] show that one of the purposes of using the search tool in ASNSs is to obtain information. He acknowledges that these sites must collect and process the information required by their users. Another study emphasizes that the researcher should use the ASNS to identify the researchers and create a cooperation network [38]. Meishar [33] stated in addition to finding information, researchers can use these sites to identify new research trends from leading researchers in various fields.  The capacity to be informative via different avenues is one of the advantages of ASNS; however, the entry of newly requested and unrelated information by the ASNS into the email and the researcher's account can be considered a weakness for the ASNS and cause the user to leave the ASNS. Customization, artificial intelligence algorithms, and user engagement in information acquisition can prevent this challenge and improve the quality of ASNS-related information.

The fifth practical feature considered by Iranian health researchers participating in the study was the possibility of facilitating the sharing and trading of laboratory materials and equipment amongst researchers. Bonaiuti [9] acknowledges that researchers can meet their needs using public posts on social media or specialized groups, which helps users of that social network find or share research resources. In the Salahshour study [29], 73% of researchers used ASNS to find material related to their research [29]. The existence of specialized groups in the ASNSs can be an effective solution for sharing laboratory materials and equipment and bolstering effective communication [9]. Given the situation in Iran and the sanctions imposed on the one hand [39] and poor economic conditions, on the other hand, this user expectation seems reasonable. Users can share the features of their laboratory materials and equipment and share their resources with other researchers. In addition to economic savings, this practice can increase research centers' efficiency and strengthen cooperation between researchers.

### Conclusions

This study aimed to identify the expectations of health researchers from ASNSs. These expectations were divided into functional and technical characteristics. Functional characteristics were related to different research processes, and researchers used these features to increase the speed and quality of their research. In this category, they expected ASNSs to facilitate communication and inform them about various research fields. Moreover, some researchers expected ASNSs to enhance the process of conducting research and help in sharing and trading laboratory materials and equipment. Managing scientific publications is a functional characteristic that includes improving and managing scientometrics and altmetrics, introducing related journals, publishing the researcher's work, raising awareness regarding the scientific ranking of other researchers, and presenting a cooperative network.

Participants' expectations of ASNSs regarding technical characteristics included user management capabilities, high security and privacy, user-friendly, and other technical features. In addition to not meeting the user's expectations of ASNS, it is abandoned by researchers in some cases due to the lack of attention by programmers to users' opinions in the design of ASNS.

## Abbreviations

ASNS: academic social network site

## References

[1] Gruzd A (2012). Non-academic and academic social networking sites for online scholarly communities. Social Media for Academics.

[2] Bik HM, Goldstein MC (2013). An Introduction to Social Media for Scientists. PLoS Biol.

[3] Khvatova T, Dushina S, Nikolaenko G (2017). Do the Online Activities of Scientists in Social Professional Networks Influence their Academic Achievements? European Conference on Management, Leadership & Governance; : Academic Conferences International Limited. ECMLG 2017 13th European Conference on Management, Leadership and Governance.

[4] Kulathuramaiyer N (2016). How Social Networks will Change Research. The IPSI BgD Transactions on Internet Research.

[5] Fiesler C, Proferes N (2018). “Participant” Perceptions of Twitter Research Ethics. Social Media plus Society.

[6] Dehghani M, Akhondzadeh S, Mesgarpour B, Ferdousi R (2020). A Tool to Reduce the Problems of Iranian Health Researchers. ijph.

[7] de Rosa A, Bocci E, Dryjanska L, Borrelli F (2016). The role of academic social networking in the dissemination of the social representations literature. 10h International Technology, Education and Development Conference, INTED Proceedings (Valencia, SPAIN, th of March, ).

[8] Krause J (2012). Tracking references with social media tools: organizing what you've read or want to read. Social Media for Academicslsevier.

[9] Bonaiuti G (2015). Academic Social Networks: How the web is changing our way to make and communicate researches. Research on education and media.

[10] Murray M (2014). Analysis of a scholarly social networking site: The case of the dormant user. SAIS 2014 Proceedings.

[11] Manca S (2018). ResearchGate and Academia.edu as networked socio-technical systems for scholarly communication: a literature review. Research in Learning Technology.

[12] Liu D, Kirschner PA, Karpinski AC (2017). A meta-analysis of the relationship of academic performance and Social Network Site use among adolescents and young adults. Computers in Human Behavior.

[13] Salvation M, Adzharuddin N (2014). The influence of social network sites (SNS) upon academic performance of Malaysian students. International Journal of Humanities and Social Science.

[14] Ortega J (2015). Disciplinary differences in the use of academic social networking sites. Online Information Review.

[15] Ghorbani N, Momeni M, Ghorbani R, Babalhavaeji F (2018). A Study on the Presence of Iranian Researchers in Academic Social Networks: A Case Study on the Faculty Members of Semnan University Of Medical Sciences, Iran. Health Information Management.

[16] Mozaffari A, Rastegari B (2015). Evaluation of The Iranian Users Trust to Privacy in Linkedin. Media Studies.

[17] Ghazimirsaeed S, Papi A, Ramezani A, YektaKooshali M, RamezaniPakpourLangroudi F (2018). Evaluation Altmetric Indicators of Iranian Medical Universities in Academic Social Networks: ResearchGate and Academia.edu. Quarterly Knowledge and Information Management Journal.

[18] Jeng W, He D, Jiang J (2014). User participation in an academic social networking service: A survey of open group users on Mendeley. J Assn Inf Sci Tec.

[19] Dehghani M, Akhondzadeh A, Mesgarpour B, Ferdousi R (2020). Design and Implementation of a Social Network for Laboratory Researchers. ircmj.

[20] Azami-Aghdash S, Ghojazadeh M, Aghaei M, Naghavi-Behzad M, Asgarlo Z (2015). Perspective of patients, patients' families, and healthcare providers towards designing and delivering hospice care services in a middle income Country. Indian J Palliat Care.

[21] Ghojazadeh M, Azami-Aghdash S, Sohrab-Navi Z, Kolahdouzan K (2015). Cardiovascular patients' experiences of living with pacemaker: Qualitative study. ARYA Atheroscler.

[22] Englander M (2016). The phenomenological method in qualitative psychology and psychiatry. International Journal of Qualitative Studies on Health and Well-being.

[23] Byrne M (2001). Sampling for qualitative research. AORN Journal.

[24] Cleary M, Horsfall J, Hayter M (2014). Data collection and sampling in qualitative research: does size matter?. J Adv Nurs.

[25] Higginbottom GMA (2004). Sampling issues in qualitative research. Nurse Researcher.

[26] Grbich C (2007). Qualitative data analysis: An introduction.

[27] Hsieh H, Shannon SE (2016). Three Approaches to Qualitative Content Analysis. Qual Health Res.

[28] Ope C, Ziebland S, Mays N (2000). Analysing qualitative data. British Medical Journal.

[29] Salahshour M, Dahlan H, Iahad N (2016). A case of academic social networking sites usage in Malaysia: drivers, benefits, and barriers. International Journal of Information Technologies and Systems Approach (IJITSA).

[30] Manca S, Raffaghelli J (2017). Towards a multilevel framework for analysing academic social network sites: A networked socio-Technical perspective. 4th European Conference on Social Media–ECSM.

[31] Aleryani A, Mofleh H, Alariki S (2017). The Usage of Academic Social Network Sites by Researchers in Developing Countries: Opportunities and Challenges. Journal of Information Technology and Networking.

[32] Bardakcı S, Arslan Ö, Ünver TK (2017). How scholars use academic social networking services. Information Development.

[33] Meishar-Tal H, Pieterse E (2017). Why Do Academics Use Academic Social Networking Sites?. IRRODL.

[34] Weber GM, Barnett W, Conlon M, Eichmann D, Kibbe W, Falk-Krzesinski H, Halaas M, Johnson L, Meeks E, Mitchell D, Schleyer T, Stallings S, Warden M, Kahlon M (2011). Direct2Experts: a pilot national network to demonstrate interoperability among research-networking platforms. Journal of the American Medical Informatics Association.

[35] Espinoza VF, Caicedo BC (2015). Academic social networking sites: A comparative analysis of their services and tools. IConference 2015 Proceedings.

[36] El-Berry DK (2015). Awareness and Use of Academic Social Networking Sites by the Academic Staff at the South Valley University in Egypt. JLIS.

[37] Dermentzi E, Papagiannidis S (2018). Academics’ intention to adopt online technologies for public engagement. INTR.

[38] Zerhouni EA (2005). Translational and Clinical Science — Time for a New Vision. N Engl J Med.

[39] Dehghani M, Mesgarpour B, Akhondzadeh S, Azami-Aghdash S, Ferdousi R (2021). How the US Sanctions Are Affecting the Health Research System in Iran?. Arch Iran Med.

